# Aneurysmal Circumaortic Left Renal Vein: A Rare Anatomical Variant Managed With Open Surgery

**DOI:** 10.7759/cureus.113570

**Published:** 2026-07-29

**Authors:** Hussein Jouni, Ali Fawzi

**Affiliations:** 1 Vascular Surgery, Hammoud Hospital University Medical Center, Beirut, LBN; 2 Vascular Surgery, Sheikh Ragheb Harb University Hospital, Beirut, LBN

**Keywords:** circumaortic left renal vein, renal vein aneurysm, saccular aneurysm, surgical management, venous aneurysm

## Abstract

Circumaortic left renal vein (CLRV) is an uncommon congenital venous anomaly resulting from persistence of both the ventral and dorsal limbs of the embryonic renal venous collar. Although usually asymptomatic and incidentally detected, its recognition is important because of its significant clinical and surgical implications. Renal vein aneurysms associated with CLRV are exceptionally rare, and their optimal management remains undefined. Here, we report a case of a 58-year-old woman who presented with chronic left flank pain of unclear etiology. Contrast-enhanced computed tomography (CECT) and renal venography demonstrated a CLRV with a 30×14 mm saccular aneurysm arising from the preaortic limb. Imaging revealed no evidence of nutcracker phenomenon, venous stenosis, thrombosis, arteriovenous malformation, or other secondary causes of aneurysm formation. Given the persistence of symptoms despite conservative management and the absence of alternative explanations for the pain, surgical treatment was undertaken. The postoperative course was uneventful, and complete symptom resolution was achieved during the follow-up period. This report describes a rare symptomatic aneurysmal CLRV successfully managed with open surgical resection. The case highlights the importance of accurate preoperative vascular mapping and suggests that surgical management may be a safe and effective treatment option in carefully selected symptomatic patients.

## Introduction

Circumaortic left renal vein (CLRV) is an uncommon vascular anomaly that is reported in 2-17% of the general population [1]. It arises by duplication of the left renal vein, with one venous channel passing anteriorly and the other passing posteriorly to the aorta, thereby encircling it [2].

Although CLRVs are usually asymptomatic and detected incidentally, their recognition is essential due to significant implications during retroperitoneal, urological, vascular, and transplant surgery. Failing to identify such variants prior to intervention may increase the risk of major vascular complications, including inadvertent venous injury, excessive bleeding, and difficulties during vascular control [3].

Previously published reports regarding CLRVs have predominantly described conservative management [3]. For instance, Lin et al. and Provenza et al. reported asymptomatic aneurysmal CLRVs that were successfully managed by serial imaging surveillance, showing no aneurysm progression or CLRV-related symptoms [4,5]. Similarly, Sakhri et al. and Wang et al. described incidental asymptomatic CLRVs identified preoperatively or intraoperatively that required no active intervention [3,6].

In contrast, we present a rare case of symptomatic aneurysmal CLRV that required definitive surgical intervention. Despite an extended trial of conservative management, the patient experienced persistent symptoms. Given the failure of non-operative measures to provide relief, open surgical repair was successfully performed. To our knowledge, this represents one of the very few reported cases where an aneurysmal CLRV presented with persistent symptoms necessitating surgical resection.

## Case presentation

**Figure 1:**
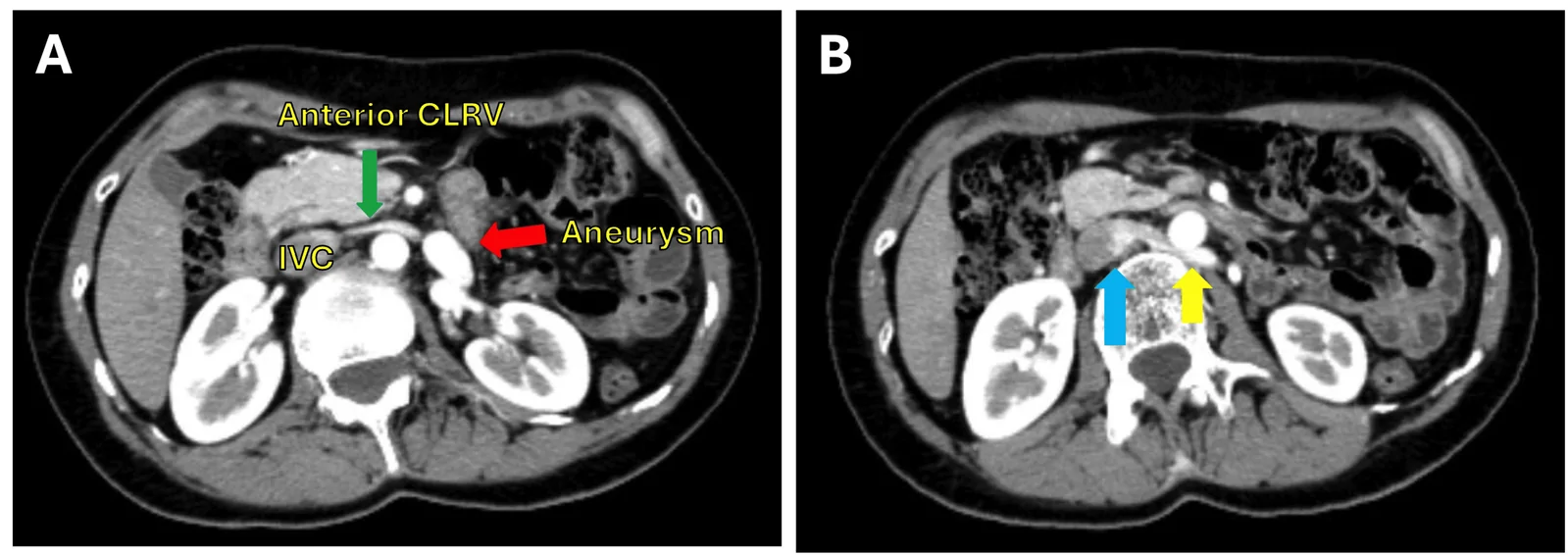
Contrast-enhanced computed tomography (CECT) of the abdomen. (A) Axial view demonstrating a circumaortic left renal vein (CLRV). The anterior limb (green arrow) courses anterior to the abdominal aorta to drain into the inferior vena cava (IVC), with an associated saccular venous aneurysm (red arrow). (B) Inferior axial slice confirming the posterior retroaortic limb (yellow arrow) passing posterior to the abdominal aorta toward its IVC confluence (blue arrow), without evidence of extrinsic compression or entrapment.

A 58-year-old woman presented with persistent left flank pain of more than one year's duration. The patient had a body mass index (BMI) of 22.5 kg/m², with no significant past medical history or family history of vascular anomalies. Despite regular analgesic use, she experienced no symptomatic relief, and the pain progressively worsened, leaving her unable to sleep on her left side. Laboratory investigations, including routine urinalysis and renal function tests, were within normal limits. Contrast-enhanced computed tomography (CECT) of the abdomen revealed a CLRV consisting of two distinct channels: an anterior limb coursing anteriorly to the abdominal aorta and a posterior limb passing retroaortic. Both limbs drained into the inferior vena cava without evidence of obstruction. Additionally, a well-defined saccular aneurysm measuring approximately 30 × 14 mm was identified arising from the anterior limb (Figure 1).

**Figure 2:**
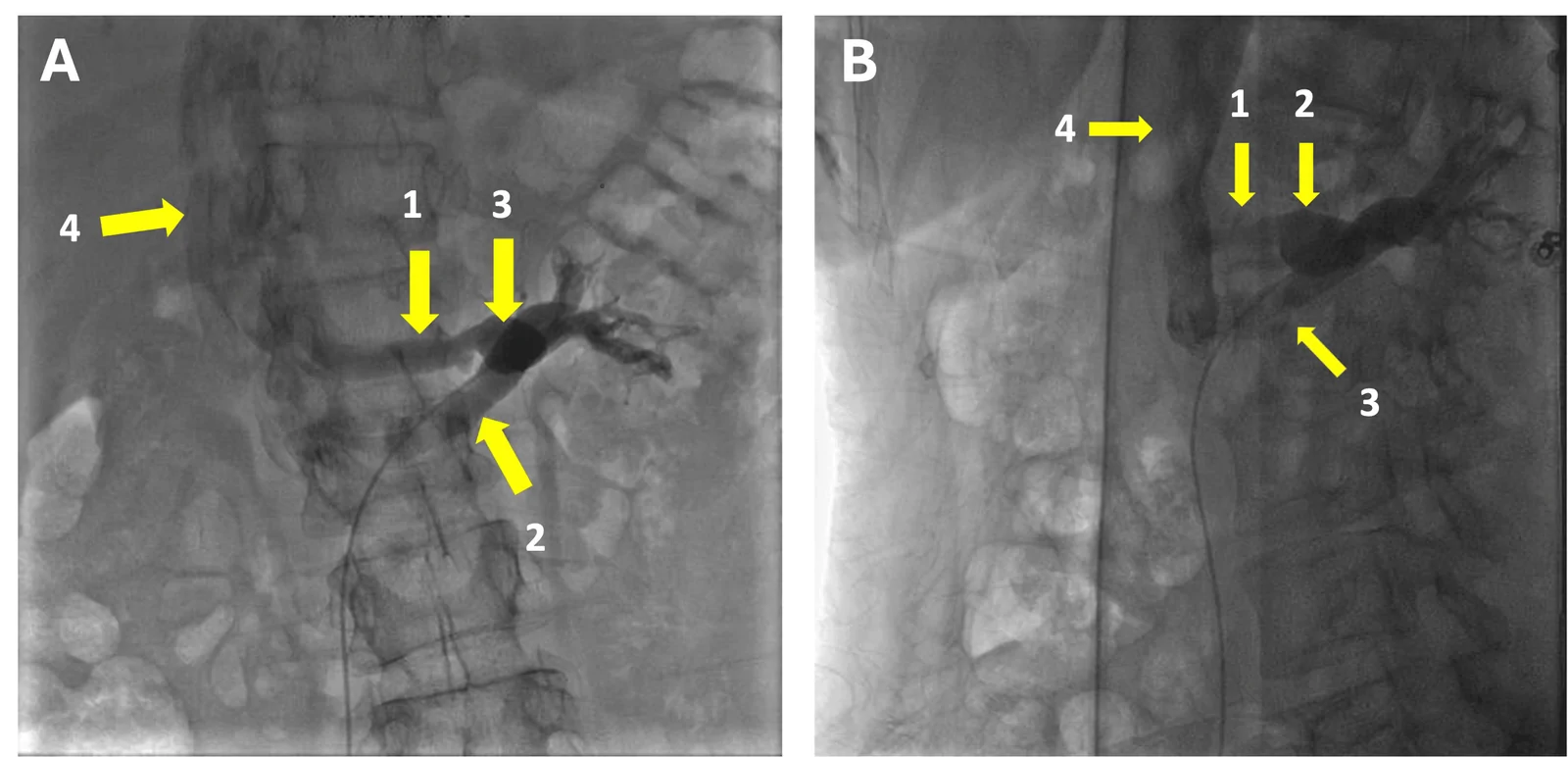
Selective venography of the left renal vein. (A) AP view: (1) anterior limb of renal vein; (2) posterior limb of renal vein; (3) aneurysmal mass; (4) inferior vena cava. (B) Lateral view: (1) anterior limb of renal vein; (2) aneurysmal mass; (3) posterior limb of renal vein; (4) inferior vena cava

Selective renal venography was performed to further delineate the vascular anatomy and lesion dynamics. Contrast injection outlined smooth borders and demonstrated rapid contrast clearance without flow stagnation or thrombosis, confirming the aneurysmal nature of the lesion. There was no evidence of intrinsic stenosis, extrinsic compression, or thrombosis in either limb of the renal vein (Figure 2).

Following a detailed discussion of therapeutic options and potential risks, the patient consented to surgical management due to persistent, debilitating symptoms and the absence of alternative etiologies. The surgical plan entailed aneurysm excision and ligation of the involved anterior venous segment, contingent upon intraoperative confirmation that the posterior retroaortic limb was patent and compression-free.

**Figure 3:**
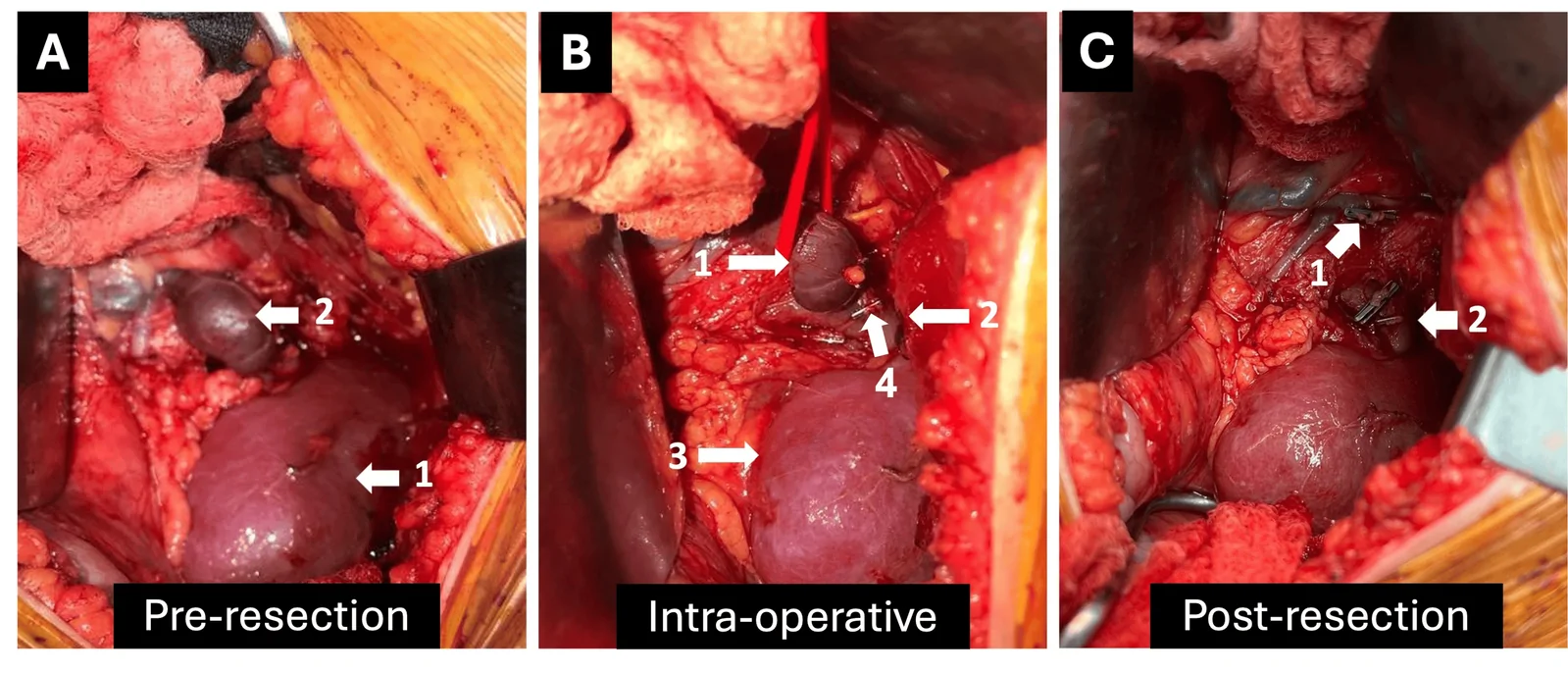
Intra-operative image of the aneurysmal mass. (A) 1: Left kidney, 2: aneurysm sac. (B) 1: Aneurysm sac, 2: posterior limb of renal vein, 3: left kidney, 4: anterior limb clamping. (C) 1: Ligated afferent limb of resected aneurysm, 2: posterior limb of renal vein

A left subcostal incision (~13 cm) was performed to access the abdominal cavity and retroperitoneum. Following mobilization of the left kidney, the anterior and posterior limbs of the CLRV collar were carefully identified. Vascular control and vessel mobilization were achieved using vessel loops placed around the involved anterior venous segments. After intravenous administration of 5,000 IU of unfractionated heparin, the tributary veins draining into the anterior limb were carefully ligated (Figure 3A). The aneurysmal segment was then meticulously dissected, isolated, and occluded using vascular clamps (Figure 3B). The posterior limb was identified and confirmed to be patent and free of compression posterior to the aorta. Given the presence of a patent retroaortic limb providing an alternative venous drainage pathway, the aneurysm was resected en bloc, and the proximal and distal ends of the anterior limb were securely ligated using surgical clips (Figure 3C). No intraoperative evidence suggested renal venous congestion or impaired drainage following anterior limb ligation. No intraoperative complications occurred. The total operative time was 90 minutes, and blood loss was minimal.

The postoperative course was uneventful. The patient was discharged on postoperative Day 3 with mild incisional pain, managed with routine oral analgesics. At the three-week and six-month follow-up evaluation, she remained completely asymptomatic with full resolution of her flank pain.

## Discussion

CLRV is an uncommon congenital vascular variant resulting from aberrant embryological development of the renal venous system. It has been reported in 2%-17% of the general population [1].

During normal embryonic development, the left renal vein originates from the renal venous collar, a network of anastomosing ventral and dorsal venous channels surrounding the abdominal aorta. Typically, the dorsal component regresses while the ventral component persists to form the definitive left renal vein coursing anterior to the aorta. Failure of the dorsal channel to regress, combined with persistence of the ventral channel, results in a CLRV collar [7].

Although CLRV is usually asymptomatic and detected incidentally, its clinical significance becomes paramount during surgical or interventional procedures. In contrast to most reported incidental cases [4,5], the present case is exceptional due to the presence of symptomatic aneurysmal dilation. The nutcracker phenomenon was excluded, as nutcracker is characterized by compression of the left renal vein between the superior mesenteric artery anteriorly and the abdominal aorta posteriorly, resulting in renal venous hypertension that may lead to venous dilatation and, in rare cases, aneurysm formation [8]. Cross-sectional imaging showed no evidence of left renal vein compression, stenosis, or associated arteriovenous malformation. Furthermore, given the absence of prior abdominal trauma or vascular intervention, the aneurysm was classified as idiopathic in origin.

CLRV exhibits considerable anatomical diversity, with various morphological patterns described in the literature [9]. Morphologically, CLRVs are classified into three major types: Type I, in which a single left renal vein bifurcates into two limbs prior to draining into the inferior vena cava (IVC); Type II, characterized by two distinct left renal veins draining independently into the IVC; and Type III, consisting of a complex network of multiple interconnected venous channels [10]. Other rare variants include veins draining into the iliac veins or displaying fenestrated patterns. Based on this classification, our patient presented with a classic Type I CLRV configuration, characterized by one preaortic limb and one retroaortic limb.

CECT and selective venography played pivotal roles in diagnosis, enabling precise anatomical mapping and confirming the aneurysmal nature of the lesion [11]. Additional imaging modalities, such as Doppler ultrasonography, can also be valuable when detailed hemodynamic evaluation is required, for example, to assess flow direction, detect renal vein entrapment in suspected Nutcracker syndrome, or monitor lesions during conservative surveillance. In the present case, CECT successfully delineated the spatial anatomy of the CLRV collar, characterized the saccular aneurysm, and ruled out extrinsic vascular compression or associated malformations, supplying essential guidance for operative planning [12,13].

The management of renal vein aneurysms is unstandardized due to the rarity of cases. Current therapeutic strategies range from conservative clinical and radiological surveillance to open or endovascular surgical intervention [5]. Surgical intervention is usually reserved for associated pathological conditions, such as aneurysmal degeneration, thrombosis, or symptomatic venous compression [3]. In surgical candidates, anomalous renal venous anatomy presents technical challenges, increasing the risk of inadvertent intraoperative venous injury, complex vascular control, and substantial hemorrhage [14].

In previously reported cases of similar anatomical variations, conservative management was predominantly selected due to the absence of symptoms (Table 1) [3-6]. For instance, Lin et al. and Provenza et al. described asymptomatic CLRV aneurysms managed safely with serial imaging surveillance over an 18-month period, demonstrating no aneurysmal growth or symptom development [4,5]. Similarly, Wang et al. reported two incidental asymptomatic CLRVs identified preoperatively, where no targeted vascular intervention was performed; neither patient exhibited symptoms attributable to the venous anomaly [6]. Furthermore, Sakhri et al. reported an incidental intraoperative identification of a CLRV during vascular surgery, highlighting the critical need for meticulous surgical dissection and preservation of the anomalous vein to prevent major iatrogenic injury [3].

**Table 1:** Published clinical reports describing the management and outcomes of circumaortic left renal vein (CLRV). *CECT: Contrast-enhanced computed tomography; CLRV: Circumaortic left renal vein

Author	Year	Clinical presentation	Management	Outcome
Lin et al. [4]	2010	Asymptomatic aneurysmal CLRV	Conservative management with serial CECT* surveillance	Stable aneurysm without progression during follow-up
Provenza et al. [5]	2022	Asymptomatic aneurysmal CLRV	Conservative management with imaging surveillance	Remained asymptomatic with no aneurysm enlargement during follow-up
Wang et al. [6]	2024	Incidental asymptomatic CLRV with double retroaortic limbs (two cases)	No intervention; anatomical recognition for procedural planning	Uneventful clinical course; no CLRV-related complications reported
Sakhri et al. [3]	2025	Incidental asymptomatic CLRV encountered during vascular surgery	Careful intraoperative identification and preservation	Successful completion of surgery without renal vein injury or CLRV-related complications
Present case	2026	Symptomatic aneurysmal CLRV	Open aneurysmectomy with venous reconstruction	Successful surgical repair with favorable postoperative recovery

In contrast, surgical intervention was pursued in the present case due to the patient's persistent and debilitating symptoms, the exclusion of alternative etiologies, and the potential long-term risks associated with venous aneurysms. Although endovascular embolization has emerged as a minimally invasive option for selected venous aneurysms, its role in visceral and intra-abdominal venous aneurysms remains poorly defined, with open surgical repair remaining the established standard of care [15]. Furthermore, endovascular embolization was deemed unfavorable in our patient case, as the high-flow hemodynamic environment of the renal vein carries a heightened risk of coil migration and catastrophic pulmonary embolism [16].

While size thresholds for intervention are well established for arterial aneurysms, definitive treatment criteria for venous aneurysms are less clearly defined [5]. Potential complications of untreated venous aneurysms include thrombosis, thromboembolism, rupture, and compression of adjacent structures [15]. Given our patient's low operative risk, persistent symptoms, and favorable anatomy, surgical resection was considered a reasonable and definitive treatment option. The successful postoperative outcome supports surgical resection as a safe and effective strategy in carefully selected patients with symptomatic aneurysmal CLRVs.

This report has inherent limitations. As a single-case report, the findings cannot be generalized to all patients with CLRV aneurysms, and definitive conclusions regarding the optimal treatment paradigm remain limited. Furthermore, while our patient demonstrated complete symptom resolution at her six-month follow-up, long-term outcome data beyond this timeframe are lacking. Due to the extreme rarity of CLRV aneurysms, standardized, evidence-based treatment guidelines do not currently exist. Future multi-institutional registries or pooled case analyses are warranted to track long-term natural histories and establish clear intervention criteria for this rare vascular variant.

## Conclusions

Renal vein aneurysms associated with a CLRV are exceptionally rare vascular anomalies. Due to the scarcity of reported cases, management strategies remain unstandardized and must be tailored to individual clinical presentations and anatomy. This case demonstrates that open surgical resection of an anterior limb aneurysm is a safe and effective treatment strategy when a patent retroaortic limb exists to maintain renal venous drainage. Comprehensive preoperative vascular imaging is essential for surgical planning to avoid inadvertent iatrogenic vascular injury during retroperitoneal interventions. As the incidental detection of complex venous variants increases with high-resolution imaging, further reporting remains vital to establish evidence-based management guidelines. Finally, further studies are needed before broader recommendations regarding the management of these cases can be made.
